# Proteome data on the microbial microbiome of grasshopper feces

**DOI:** 10.1016/j.dib.2016.11.033

**Published:** 2016-11-16

**Authors:** Nico Jehmlich, Martina Müller, Stefanie Meyer, Alexander Tischer, Karin Potthast, Beate Michalzik, Martin von Bergen

**Affiliations:** a Department of Molecular Systems Biology, Helmholtz Centre for Environmental Research – UFZ, Permoserstr. 15, 04318 Leipzig, Germany; b Institut für Geographie Professur Physische Geographie/Schwerpunkt Bodenkunde, Löbdergraben 32, 07743 Jena, Germany; c Aalborg University, Department of Chemistry and Bioscience, Fredrik Bajers Vej 7, 9220 Aalborg, Denmark; d Faculty of Biosciences, Pharmacy and Psychology, Institute of Biochemistry, University Leipzig, Leipzig, Germany

**Keywords:** Metaproteomics, Microbiota, Feces, Grasshoppers

## Abstract

We present proteome data from the microbiota (feces) after a diet shift from a natural diverse to a monocultural meadow with *Dactylis glomerata.* The abundant grasshopper species, *Chorthippus dorsatus,* was taken from the wild and kept in captivity and were fed with *Dactylis glomerata* for five days. For phytophagous insects, the efficiency of utilization of hemicellulose and cellulose depends on the gut microbiota. Shifts in environmental and management conditions alter the presence and abundance of plant species which may induce adaptations in the diversity of gut microbiota. The mass spectrometry proteomics data have been deposited to the ProteomeXchange Consortium via the PRIDE partner repository with the dataset identifier PXD005126.

**Specifications Table**

**Table:** 

Subject area	Biology
More specific subject area	Metaproteomics
Type of data	1) Mass spectrometry data (*.raw) 2) Search output data (*.msf) 3) Figures (PowerPoint files)
How data was acquired	Orbitrap Fusion mass spectrometer (Thermo Scientific) coupled with the TriVersa NanoMate (Advion Biosciences, Norwich, UK).
Data format	1) msf (Proteome Discoverer output files) 2) pptx (PowerPoint files)
Experimental factors	Microbial proteins were isolated form feces, proteolytic cleaved using trypsin and subsequently analyzed by LC-MS/MS
Experimental features	1) Grasshopper feces collection 2) Protein extraction 3) LC-MS/MS analysis
Data source location	Leipzig, Saxony, Germany
Data accessibility	Data is within this article. The mass spectrometry proteomics data have been deposited to the ProteomeXchange Consortium via the PRIDE partner repository with the dataset identifier PRIDE: PXD005126.

**Value of the data**

• Protein assessment of the microbiota of the grasshopper species *Chorthippus dorsatus*.
• Metaproteome from the grasshoppers provides the basis for more functional analyses of the grasshopper microbiota.
• Relevant information for the grasshoppers ecology on the basis its microbiota.

## 1 Data

We present the first dataset of this relevant type of phytophagous insects since there is so far no metaproteome dataset on the gut microbiota of grasshoppers available. To detect the diet dependent metabolic adaptation, a shift in diet from a diverse food source to the single-species food *Dactylis glomerata* was performed.

## 2 Experimental design, materials and methods

### 2.1 Grasshopper culture and feces sampling

We selected *Chorthippus dorsatus* that was at that time the most dominant grasshopper species on a ruderal meadow that has been under this type of land use for 20 years. The meadow was dominated by grasses, namely *Poa pratensis, Dactylis glomerata*, *Festuca pratensis*, and *Bromus sterilis*, which are all potential food plants for the generalist herbivores in grasslands [1], [2], [3], [4]. Three days before the start of the experiment, we caught female and male grasshoppers with sweep nets and kept them separately in cages with a mixed grass diet from the meadow until the start of the experiment. In August 2014, 2 male and 2 female grasshoppers were transferred to a cage without food for 1 day to synchronize for gut content. On the following day *D. glomerata*, which was grown in a climate chamber under standardized and controlled soil and moisture conditions for about 2.5 months, was added to the cage. Excrements were sampled before (d0) and after adding *D. glomerata* at day 1, 3, and 5 (day 1, day 3 and day 5) for one week and frozen at −80 °C.

### 2.2 Protein extraction and sample preparation

After the sampling of feces (day 0, 1, 3, 5 and 6), three feces were pooled and were considered as one replicate. For protein extraction, to the feces 4 glass beads (3 mm, Carl Roth GmbH), two spatula tips of zirconium beads (0.1 mm diameter, Biospec.) and 800 µL of lysis buffer (500 mM NaCl, 50 mM Tris–HCl, pH8, 50 mM EDTA, 4% (w/v) SDS) were added. Feces were disrupted by FastPrep (3×1 min, 5.5 ms^−1^, MP Biomedicals) and incubated for 15 min at 95 °C. After centrifugation (14,000 rpm, 5 min, 4 °C), the supernatant was taken. To the pellets 300 µL of lysis buffer were added and the procedure with FastPrep and heating was repeated exclusively for the pellet. The supernatant was precipitated over night at −20 °C with acetone (2.5 fold of ice-cold acetone). Protein pellets were harvested by centrifugation (14,000 rpm). Dried pellets were dissolved in 20 µL of SDS sample buffer (2% w/v SDS, 2 mM beta-mercaptoethanol, 4% v/v glycerol, 40 mM Tris–HCl pH 6.8, 0.01% w/v bromophenol blue), heated to 90 °C for 4 min and separated by SDS polyacrylamide gel electrophoresis. Proteins were stained in gel with Coomassie G-250 (Merck). Gel was cut into small pieces (band per samples), destained, dehydrated and proteolytically cleaved overnight at 37 °C trypsin (Promega) [5]. Extracted peptides were desalted using C18 ZipTip column (Merck Millipore). Peptide lysates were re-suspended in 0.1% formic acid and injected to liquid chromatography mass spectrometry (LC-MS/MS).

### 2.3 Mass spectrometric measurement and data analysis

**Fig. 1:**
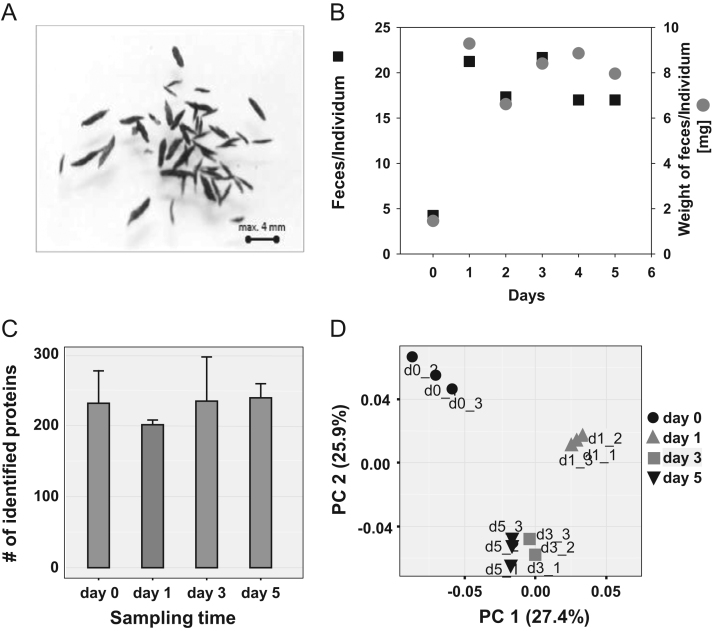
Feces and protein identification. (**A**) Representative image of grasshopper feces as they are collected after each day. (**B**) Scatter plot of the number of feces and weight of feces divided by the number of grasshoppers. The feces are shown as a black square and the weight is indicated in gray circle. (**C**) Principal component analysis (PCA) plot of the identified bacterial proteins along the time-points. Each time point was analyzed in triplicates. Time point d0 represents one day without feeding (black circle), time point d1 is the first day of feeding on *Dactylis glomerata* (gray triangle), time point (d3) as a gray square, time point (d5) as a black triangle and time point (d6) as a black square. The first two components account for about 53% variability within the proteomic dataset. (**D**) Bar chart of protein identification for each time point. The number of protein identifications range from 201 to 239 for individual measurements. Most protein groups were identified at the time point (d5) (239 protein groups) and least at the time point (d1) with 201 protein groups. Error bars show the standard deviation between triplicate measurements.

**Fig. 2:**
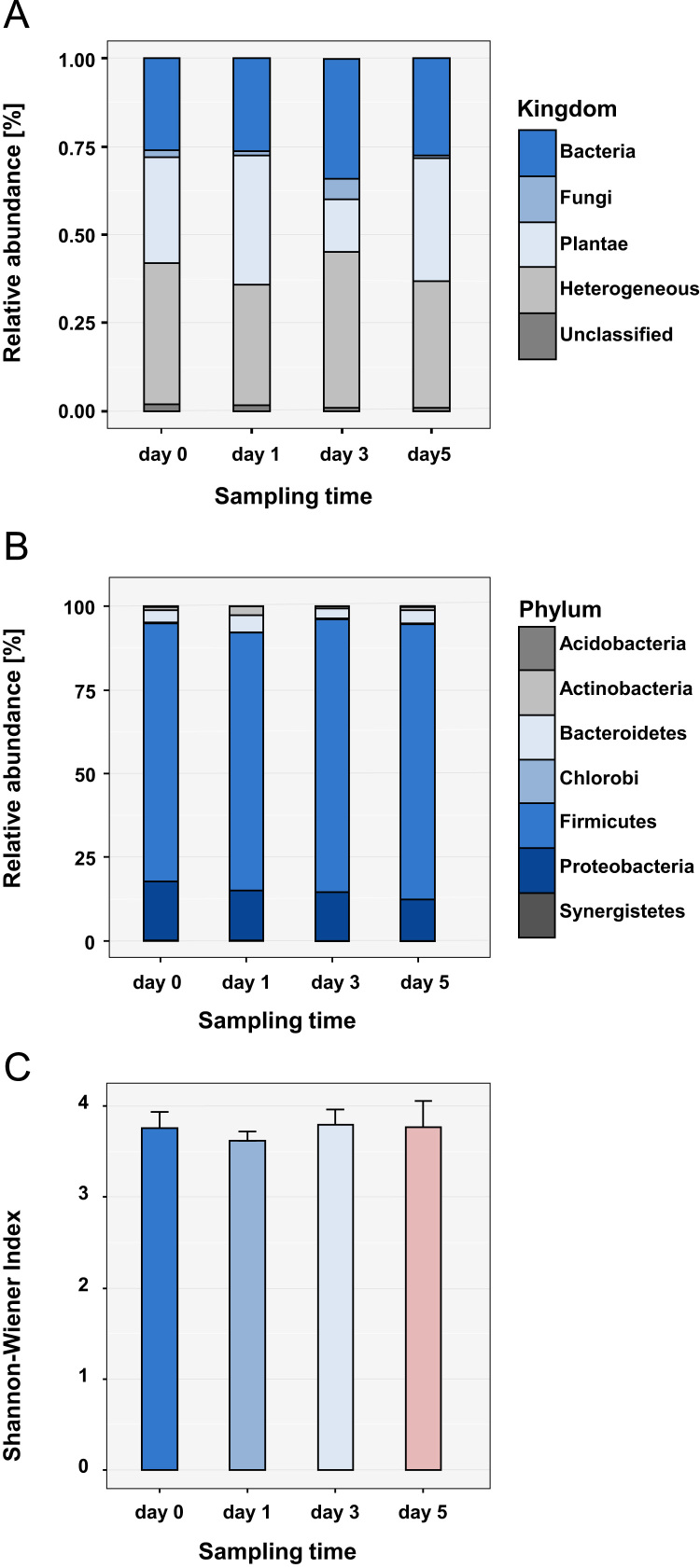
Phylogenetic resolution of identified proteins. (A) Stacked bar chart show the distribution of identified protein groups assigned to phylogenetic kingdoms. The two most abundant groups along the time points belong to kingdom Bacteria (in dark blue) and kingdom Plantae (in light blue). (B) Phylogenetic distribution at the phylum of Bacteria. 77% to 82% of all bacterial proteins were classified to phylum Firmicutes. The second most abundant phylum (range from 12% to 17%) was Proteobacteria. (C) Bar chart of bacterial alpha diversity over the time course. Error bars show the standard deviation between triplicate measurements. There were no significant differences of alpha diversity observed.

**Fig. 3:**
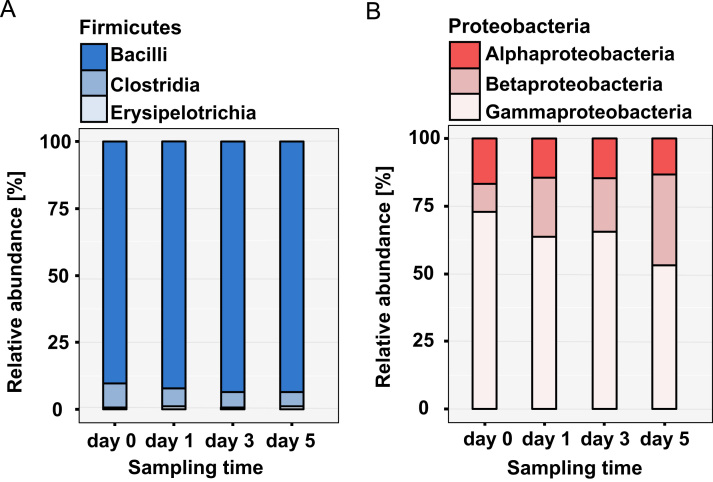
Phylogenic resolution for the two most abundant phyla. (A) Stacked bar chart in blue colors shows the representation of phylum Firmicutes. The class Bacilli (in dark blue) were along the time points the most presented (over 90% of all Firmicutes proteins). The next bacterial classes presented in phylum Firmicutes were class Clostridia (5–9%) followed by Erysipelotrichia (0.7–1.2%) (B) Stacked bar chart (in red colors) shows the second most abundant phylum Proteobacteria. Class Gammaproteobacteria (in light rose) was the most abundant class of Proteobacteria. And their abundance decline from 73% at time point d0 to 53% at time point d5. The number of identified proteins from the class Betaproteobacteria increase from 10.4% at time point (d0) to about 34% at time point (d5). Alphaproteobacteria (in deep red) remained over the time points at the same level (13–17%).

**Fig. 4:**
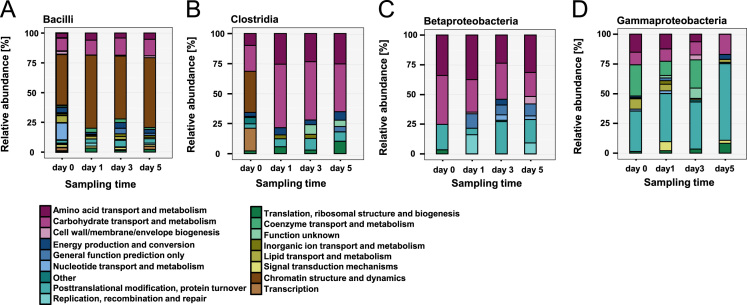
Functional groups (by cluster of orthologous group (COG) categories) identified by most abundant taxonomic classes. (A) Stacked bar charts show the protein classification of proteins into 17 most common functional groups. Proteins from class Bacilli assigned to functional groups. The most abundant functional group is *Chromatin structure and dynamic* which increase from time point (d0) (43%), the time point (d1) (61%) to time point (d6) (59%). The next group is *Amino acid transport and metabolism* and *Carbohydrate transport and metabolism* at constant abundance (4–6% and 11–15%). (B) Proteins from class *Clostridia* matched to functional groups. *Chromatin structure and dynamic* is present at time point (d0) with 34%, but disappeared at the later time points. *Carbohydrate transport and metabolism* increases from d0 (21.7%) to d1 (53%) and then slowly decrease to d6 (44%). *Amino acid transport and metabolism* has also increase of the abundance from d0 (10%) to d6 (44%). (C) Betaproteobacteria. *Carbohydrate transport and metabolism* decrease from d0 (41%) to d5 (20%). *Amino acid transport and metabolism* is rather dynamically (d0 34%, d1 37%, d3 30%, d5 32% and d6 53.3%) but become prominent at the later time point. (D) Gammaproteobacteria. *Carbohydrate transport and metabolism* accomplished by Gammaproteobacteria remain rather stable (d0 11%, d1 10%, d3 11%, d5 17%, d6 16%). *Amino acid transport and metabolism* slowly decrease from d0 (15%) to d5 (6%) and then increase to d6 (17%). *Posttranslational modification, protein turnover, chaperones* increase from d0 (34%) to d5 (65%). *Coenzyme transport and metabolism* is also presented in higher abundance (d0 26%, d1 12%, d3 24%, d5 0% and d6 5%).

**Fig. 5:**
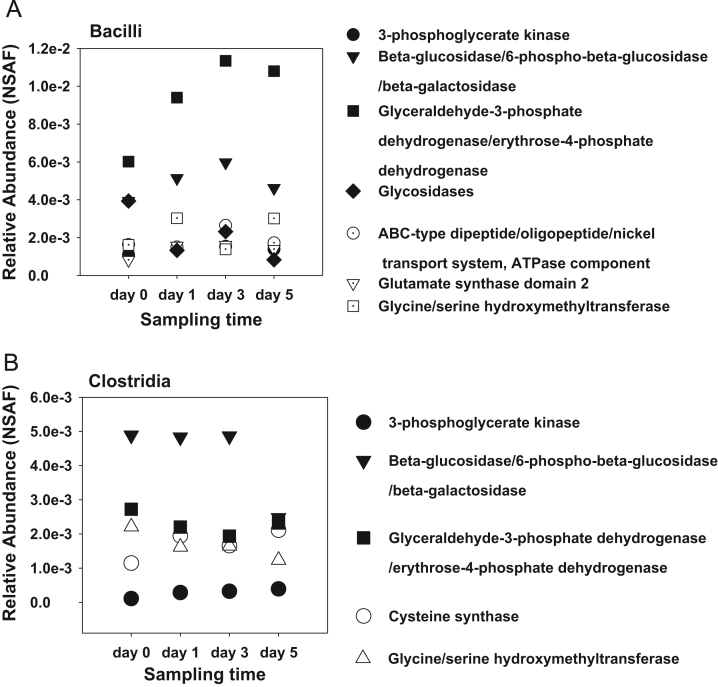
Relative protein abundance levels. Protein abundances of selected proteins assigned for (A) Bacilli and (B) Clostridia were calculated based on the normalized spectral abundance factor (NSAF) and plotted along the time-line in order to observe species abundance changes in respect to their functional classification.

Mass spectrometry was performed using Orbitrap Fusion (Thermo Fisher Scientific) coupled to a TriVersa NanoMate (Advion) as described [6]. To assess the protein functions of the different bacterial community members, we assigned the identified proteins to clusters of orthologous groups (COGs). A principal component analysis (PCA) was applied as classical means of dimensionality reduction and visualization of multivariate data. PCA was assessed of log-transformed and normalized protein abundance profiles along the time scale (Fig. 1, Fig. 2, Fig. 3, Fig. 4, Fig. 5).
